# Long Term Follow up of Young People With Chronic Fatigue Syndrome Attending a Pediatric Outpatient Service

**DOI:** 10.3389/fped.2019.00021

**Published:** 2019-02-21

**Authors:** Katherine S. Rowe

**Affiliations:** Department of General Medicine, Royal Children's Hospital, Melbourne, VIC, Australia

**Keywords:** chronic fatigue syndrome, ME/CFS, adolescent, follow up study, child, functional outcome, duration of illness

## Abstract

**Aim:** To determine the reported duration of illness, the functional and educational long-term outcomes, predictive factors for recovery and seek feedback regarding management in pediatric/adolescent myalgic encepahalomyelitis/chronic fatigue syndrome (ME/CFS).

**Methods:** A cohort observational study of 784 young people, mean age 14.6 (6–18) years, with ME/CFS diagnosed at a specialist pediatric hospital and receiving regular care, was conducted with follow-up for a mean 8 (range 1–21) years after onset. Baseline symptoms, history, depression and anxiety questionnaires were available from 418. The remaining 366, did not have similar standardized baseline information. Questionnaires requested functional rating, persistent symptoms, duration of illness if “recovered,” social engagement and school/work attendance. Feedback was sought regarding management, support services, useful information, helpful interventions or personnel and use of alternative therapies. Reported recovery and function were compared with baseline information and between the two groups.

**Results:** Follow-up data were returned from 81.8%. There was no significant difference in functional score (if reported recovery) or illness duration related to provision of baseline data. The mean duration of illness was 5 (range 1–15) years in the 50% who reported recovery. By 5 years 38% and by 10 years 68% reported recovery. At 10 years the mean functional score was 8/10 (range 2–10) with 5% scoring <6. Depression, anxiety or severity of illness at diagnosis was not predictive of non-recovery. Designing and monitoring their own management plan that included educational, social, physical and enjoyable activities, as well as having symptom management and understanding professionals were highly valued. However, remaining engaged in an education system that flexibly accommodated their illness and aspirations was consistently reported as crucial for long term functioning.

**Conclusions:** ME/CFS in young people has a mean duration of 5 years (1–15) with 68% reporting recovery by 10 years. All improved functionally with 5% remaining very unwell and a further 20% significantly unwell. There were no obvious baseline predictors for recovery. However, depression, anxiety, orthostatic intolerance and to a lesser extent pain at follow up were identified as hampering recovery or function. Supportive professionals, remaining engaged in education and management strategies were identified as helpful.

## Introduction

Questions that either parents or young people ask soon after receiving a diagnosis of Myalgic Encephalomyelitis/Chronic Fatigue Syndrome (ME/CFS) are: “how long will the illness last”; “is there way of telling whether they will recover,” and “what can be done to manage the illness in the best way possible?”

ME/CFS is a condition of unknown etiology that commonly follows an infection in young people. There is a new onset of fatigue that has been present for at least 3–6 months and is not relieved by rest and not explained by other medical conditions. Post-exertional malaise, cognitive difficulties, unrefreshing sleep are present, in addition to a variety of somatic symptoms such as pain, (headache, abdominal or muscle pain), as well as flu-like symptoms without high fever and often symptoms associated with orthostatic intolerance.

Challenges to providing answers to these questions include: confirming that those followed up have a diagnosis of ME/CFS not just fatigue; ensuring that the maximum proportion of those who are diagnosed are included in the follow up; having a range of severity at diagnosis; and having an adequate cohort size with regular follow up, for sufficient length of time, in order to be confident that a realistic estimate has been obtained. Retention rates in follow up studies may also be affected by those who recover or those who remain very unwell, who may not wish to remain in contact with the medical profession. Hence perceptions of outcomes remain distorted. In addition, young people in early adult life are mobile, move away from home and frequently change their name, or transition to adult services, so following up a pediatric cohort has added challenges.

Carter et al. (1), describe outcomes for “chronic fatigue” in adolescents. However, this was at a time when ME/CFS was not well recognized in young people. Norris et al. (2) use the term “chronic disabling fatigue” identified from a survey and without clinical assessment, for their outcome study. Similarly Rimes et al. (3) used repeated community surveys to both diagnose fatigue and chronic fatigue syndrome for their estimates of outcomes but the small yields mean the findings are difficult to interpret. Krilov et al. (4) indicated that half the cohort had fatigue for only 1–6 months when first seen and 70% were followed up for 1–4 years afterwards to provide their estimate of duration of illness. Gill et al. (5) followed 34 (69% of cohort) who were retrospectively diagnosed with CFS or idiopathic fatigue for up to 4.5 (1–8) years. Van der Werf et al. (6) followed a cohort of young people with short duration of illness for 12 months. However, Bell et al. (7) followed 35 (76%) of a well-defined group for 13 years. Hence information regarding outcomes in ME/CFS in young people is scarce due to differentiating chronic fatigue from ME/CFS and ensuring that sufficient numbers are followed for an adequate period of time.

Differences in how recovery is reported and what that means to the young person add other variables. Measures of fatigue (8), symptom presence and functional outcomes, (9, 10), self-report (6), or a combination of global functioning and self-report have been used (7) thus making comparisons difficult. In describing a 2 year follow up of 54 young people, van Geelen et al. (9) reported that 50% improved in symptoms but the majority were reportedly missing more than one third of school. Hence it is important to document function and symptoms as well as perceptions of recovery. Parslow et al. (11) have been investigating what aspects of defining recovery are important to young people with ME/CFS. Understanding what a young person considers as “recovery” in a chronic illness can be problematic, but even more so in a condition that impacts every aspect of their development (social, educational, physical, and emotional) during adolescence. Without careful consideration of these aspects of development, it can be very difficult to interpret outcomes meaningfully.

Although most studies have attempted to assess the natural history of the condition, other studies have reported long term outcomes following interventions. Five year follow-up post-trial of intravenous immunoglobulin in young people (12), indicated that the mean functional outcome of the placebo group at 5 years matched that of the intervention group 6 months after the final infusion suggesting a significant number had recovered by that time (13). Other interventions that have been helpful (14) and have assisted with improving function, have not necessarily reported “recovery” (15). There have not been other convincing treatments that have altered the course of the illness (10). Although a variety of management strategies such as adaptive pacing, graded exercise, cognitive behavior therapy (14) can be helpful, the evidence for significant improvement is scarce and hampered by difficulties in comparing outcome measures according to clinical presentation, patient characteristics, case criteria and degree of disability (16). Managing some of the co-morbid or contributing conditions such as orthostatic intolerance (17) has also modified function. However, follow up after these interventions has often been for a short period of time, so realistic estimates of the duration of chronic illness is problematic.

This study sought to provide answers to the three common questions. How long does it last? Is there any way of predicting how long it will last, and what is helpful from the young person's viewpoint in managing the illness?

This study is an observational cohort study of 784 consecutive patients, diagnosed with ME/CFS after referral to an outpatient service at a specialized pediatric tertiary referral hospital, who received supportive medical care and were followed for a mean 8 (range 1–21) years.

The aims of this study were: (1) to document the long term functional and educational outcomes of a cohort of 784 young people with ME/CFS, 418 of whom provided standardized baseline medical and psychological information and additional follow up data on up to 6 occasions, 2–16 years after diagnosis; (2) to determine the duration based on self-reported recovery including the proportion reporting recovery at 5 and 10 years; (3) to identify any predictors of recovery based on baseline information obtained, and (4) to obtain feedback regarding useful management.

The objectives of the study were: (1) to describe the demographic, medical and psychological characteristics of the cohort, including the medical management; (2) to follow up the entire cohort for reported recovery, functional and educational outcomes and feedback regarding management; (3) to compare the functional and reported recovery between the group providing baseline data and the group that did not, to determine if there was a systematic bias; (4) to investigate whether there is an association between questionnaire identified depression and anxiety, or antinuclear antibody presence and reported recovery; and (5) to obtain feedback regarding management.

## Methods

### Characteristics of the Cohort and Description of Routine Medical Care

#### Setting

The Royal Children's Hospital is a specialized secondary and tertiary referral pediatric and adolescent hospital that services metropolitan Melbourne and all rural areas for the state of Victoria including bordering areas in neighboring states. Furthest distances require 4–5 h of car travel. Referrals are received from family doctors or from specialist pediatricians. Victoria has a population of 5.8 million and is multicultural with successive waves of immigrants or refugees from different parts of the world contributing to the mix. There is a universal health system that ensures citizens can access health care free of charge to the family. Twenty-six per cent of its citizens are born overseas from 200 countries, speaking 260 different languages, with an additional 30% having at least 1 parent born overseas (18). The hospital clinics reflect this demographic. More than 70 languages are spoken in the hospital.

#### Chronic Fatigue Syndrome Clinic

The clinic has been functioning since 1989. In the early years of the clinic, the Holmes definition and Fukuda criteria for CFS were available (19–21). However, acceptance of the diagnosis in young people was uncommon in the medical fraternity, and it was uncertain if the illness was similar to that in adults. Nonetheless, it was well recognized that Epstein Barr Virus (EBV) infection (or glandular fever) could run a prolonged course during adolescence with comparable symptoms. Irrespective of whether EBV was confirmed, it was assumed in some cases, that this was the cause. Alternative explanations that were often entertained were depression, “stress,” school refusal or somatization disorder or the possibility of undisclosed family difficulties. Parents who were anxious due to concern about the unexplained change in the young person were often considered to be contributing to their illness. Hence many who attended the clinic had experienced unsatisfying encounters with the medical profession.

Accurate documentation of the presentation was therefore important as described below. Ninety two patients diagnosed in the first 5 years using Holmes et al. (19) definition participated in an intravenous immunoglobulin trial (12) and were followed up separately every second year for a 5–year follow up post-trial (13) who were not included, but used for comparison in this study. Symptom patterns and characteristics of the patient group for the first 189 have also been reported elsewhere (22). Those analyses indicated that reported symptomatology was very consistent among the young people in this population, and the presence of post-exertional malaise (PEM), unrefreshing sleep, cognitive difficulties, persistent fatigue and pain (headache, muscle, abdominal) were all almost universally reported. Sore throats and glands, feeling hot and cold, and symptoms later recognized as associated with orthostatic intolerance were very common.

As immunoglobulin was a scarce resource requiring approval by a government agency, a decision was made to not allow intravenous immunoglobulin to be available for ME/CFS for young people due to some adverse effects (12), as well as inconclusive trials in adults (23–25). Thus, options for treatment reverted to general management strategies for chronic illness. We relied on feedback from young people to inform us regarding what was helpful in their management. The service has since expanded to several pediatricians and access to a 4-week self-management program run by the Victorian Pediatric Rehabilitation Service at the hospital.

#### Participants

A diagnosis of Chronic Fatigue Syndrome was made in 784 young people following an extensive history, examination, and routine investigations to exclude alternative diagnoses and confirm the presence of key symptoms identified from the earlier study (22). PEM, unrefreshing sleep and cognitive symptoms were required in addition to the Fukuda criteria (20). Other conditions including school refusal, somatization disorder, eating disorders, isolated significant depression or anxiety, connective tissue disorders, celiac disease or endocrine disorders were specifically checked. An adolescent psychosocial (HEADSS) screen was also conducted where appropriate (26). Passive standing test was not routinely performed initially. However, upon recognition of the association of orthostatic intolerance with ME/CFS this assessment was included.

Routine screening investigations included celiac screen, thyroid function and antinuclear antibody. The laboratory reported antinuclear antibody titers of 1:40 and above until 2008, when titers of 1:160 or above were considered significant. Serology for EBV or Cytomegalovirus (CMV) was routinely assessed or if there was any likelihood of overseas or tropical infections, or being in areas where Ross River Virus, Q fever (coxiella burnetti), Barmah forest virus were endemic, serology for exposure was also checked.

#### Ethics and Informed Consent

Institutional ethics approval was obtained for the baseline questionnaires and informed consent was obtained from the young person. For the follow up study, ethics approval was obtained to contact the last known address to obtain consent to forward a questionnaire for feedback on management strategies, progress of the illness and rates of reported recovery. If the young person was still at home their verbal consent was obtained or if their parents were still at the address they either provided a forwarding address or offered to forward the questionnaire which included documentation for informed consent. Occasionally the family was contacted from information in the national or state telephone directory.

#### Baseline Information

The baseline demographic and symptom questionnaire used by Lloyd et al. (25) and Rowe (12) (Appendix 1) in their immunoglobulin trials, was given to 489 of the 784 young people (mean 14.8 age 7–18) (with 55 concurrently receiving the De Paul Pediatric questionnaire). This included type of onset, family history and presence of co-morbid conditions. In addition, scales for depression (27, 28), anxiety (29), General Health Questionnaire (30) and Parental Bonding Questionnaire (31) were completed. Initially the Child Behavior Checklist (Achenbach) and Family environment scale (Moos) were included, but discarded as noncontributory after assessing 150 young people. The Parental Bonding Questionnaire was also not included after the CFS group showed no difference in scores with a matched community sample. Any abnormal laboratory findings were documented. The symptom pattern, severity and frequency were compared with questionnaire responses from an earlier separate sample (22) from the same clinic to confirm consistency in reported symptoms across time. The baseline information was collated using descriptive statistics (Statistica 13 -Statsoft –TIBCO).

#### Routine Care Offered at the Clinic

##### Initial appointment

Following diagnosis, the young person was asked: to rate the most troublesome symptom/s that he/she would like help with; to outline his/her aspirations prior to illness; to describe current school attendance, interests, and previous participation in sport, the family situation and supports including parental work schedule, and means of transport to school or activities. The young person was provided with a brief explanation of our current knowledge, a plan for managing the most severe symptoms, and an outline of a management plan that the young person would devise.

##### Management plan designed by the young person

The rationale for the management plan was to minimize the impact of chronic illness while accommodating the specific issues associated with ME/CFS. As ME/CFS affects the educational, physical, social and emotional aspects of their life, it was considered important to not neglect any of these areas. This should include some proactive social contact, academic input, physical activity and a commitment to attend something enjoyable outside of home on a regular basis. None of these activities was to be neglected but the proportion did not have to be equal. The plan needed to be sustainable for at least a month before it was reviewed. For example, some physical activity is required to prevent becoming so de-conditioned that they were not sure whether they were weak and fatigued because they were unwell or because muscles weren't being used. Social contact is important to ensure that the social learning that occurs during adolescence (how to respond in different situations, what behavior is acceptable and how to interpret different social situations and how to understand one's peers) is not neglected. It can be very daunting later when it is expected that these skills have been acquired. Academic engagement is important so that they feel that their life chances have not been destroyed. The regular enjoyable activity outside of home is something that they have chosen to go to because it is “worth it” and will not result in a prolonged recovery. It removes any prevarication regarding whether they feel well enough, whether they would cope or whether it would be easier not to go. Only if they are unable to move out of bed do they not attend. This hopefully prevents the reluctance to make decisions, to be adventurous or to be reliable.

In addition, young people generally have not had to learn to prioritize their activities during their teenage years but this skill is needed as developing adults. It is explained that they need to acquire this useful skill much earlier than most. Some activities, for example, attending school for an enjoyable subject could fulfill social, academic, and enjoyable activities and also require some physical activity. If their important social network was outside of school then there needed to be an effort to engage with that group for a period of time that was manageable. If some young people felt that “life was not worth living” if they couldn't play sport, as this was their main social connection, then adjustments could be made so that they could be part of the team by “coming off the bench” for a few minutes or not having a requirement to actively train, or else they could be moved to a team position that did not require a lot of stamina. On the other hand, for some, physical activity may be a few activities of daily living spaced over the day, or once they are able to do some activity and have increased their strength they often chose a variety of activities that they enjoy.

Their aspirations (prior to becoming unwell) played a key role in the decisions regarding their education. Attending school for set hours, rather than for specific subjects was difficult to sustain. Reduction in the school subject load to include subjects and teachers they liked, as well as subjects that were pre-requisites for what they wanted to do as a career was crucial. Trying to keep up with all subjects when only minimal information was given was a source of unnecessary stress, and this rarely succeeded. A planned timetable ensured that the arrangements could provide some consistency and predictability for the family and be manageable for the young person. If the symptoms were severe, the extent of “academic input” may be reduced to reading about a hobby or reading a story that they were already familiar with.

It was explained to the young person that these consequences of illness can be more damaging than the illness itself and can occur with any chronic illness. Neglecting these areas creates significant hurdles to recovery such as: navigating social anxiety and social learning; entering the workforce without a potentially enjoyable, satisfying or more lucrative, less physically demanding job; needing to increase strength, or not having the confidence or resilience to know how they are able to manage their life. The young person was asked to estimate how this could be achieved within the bounds of the amount of energy available over the period of a week. The young person was to make the decisions over the next few weeks and to discuss their plan with their parents.

##### Symptom management

Due to concerns with medication in young people and the risks of multiple medications, only the most severe one or two symptoms were treated. Often treating one symptom such as sleep disturbance, and allowing them to take control of their life with the management plan reduced the severity of some of the other troublesome symptoms. Despite the prominent fatigue, malaise and concentration difficulties, the complaints of headache and sleep disturbance or dizziness and nausea due to orthostatic intolerance, could often be managed effectively.

Difficulties with sleep initiation, sleep phase shift, frequent waking and disturbing nightmares were actively managed with sleep hygiene techniques and melatonin or low dose tricyclic medications such as dothiepin or amitryptiline. Simple migraine prophylactic medications such as pizotifen or periactin were anecdotally effective in reducing the severity of headache and simple measures with increasing salt and fluid including electrolyte drinks and encouraging lower limb exercises and gentle exercise could assist with orthostatic intolerance. Similarly, muscle pain and fibromyalgia could be helped by reducing sleep disturbance and encouraging gentle exercise or physical therapy.

Residual difficulties with concentration, recognition of depression, persistent severe dysmenorrhea associated with exacerbation of CFS symptoms, ongoing nausea, abdominal discomfort, persistent orthostatic symptoms were addressed after review and the implementation of the management plan.

##### Review appointments

A 6-week follow up appointment was scheduled for review of their plan, including whether the logistics were sustainable, to check on residual symptoms, and whether the symptom management was appropriate. Any further queries from the young person were addressed. Once a decision had been made regarding the schedule for education, appropriate explanation, documentation, advocacy, extra support, special provision or special consideration and tailoring a specific education program to ensure maximum possible opportunity to participate, was provided or requested from the education authorities. Sometimes there was a combination of Distance Education and school attendance for 1–2 subjects, or attendance for a few classes with Visiting Teacher assistance. If necessary, the minimum requirements were negotiated to ensure the year level was passed and that they could progress with their peers. Additional details regarding educational strategies used by the Visiting Teacher Service have been documented (32). If adjustments to sport schedules were required, these were provided and coaches and staff were usually very accommodating once they understood the reasons for the requests.

Generally 3-monthly reviews were arranged to assess progress, educational issues, symptom management and review of goals. They were seen more frequently if necessary. Occasionally young people were followed up by a local pediatrician.

In addition, parents often needed help navigating the difficult adolescent period and uncertainties regarding assisting with the tasks of adolescent development in the context a chronic illness that is generally not well understood. Parents are not sure if they should be defending, protecting and trusting the young person's judgment, or cajoling, setting limits and allowing the young person to make mistakes. Many parents had put their life “on hold” to care for the young person with the attendant complications for the whole family, and this often added significant stressors. For many young people doing some small chores that did not require much effort was important to be part of the family and reduce tensions with siblings.

### Follow Up Study

#### Participants

The cohort of 784 (mean age 14.8, range 6–18 years) diagnosed with ME/CFS over a 20 year period from 1991 was followed up between January 2008 and June 2011 to document reported outcomes. Initial contact via last known address, parental contact or national telephone directory provided verbal information and consent for forwarding questionnaires.

#### Content of Questionnaire

The follow up questionnaire (Appendix 2) asked about proportion of work or school attended, use of educational support, Visiting Teacher service, disability support, educational level achieved, illnesses experienced, exacerbations of ME/CFS, reported recovery and duration of illness if recovered. Feedback was sought regarding useful or helpful information, useful or helpful personnel (medical or otherwise), use of alternative therapies and their perceived effectiveness, and whether anything could have been handled better during their illness. Any family history of ME/CFS was also asked. Both the Bell CFIDS Disability Scale (33) and a global rating 1–10/10 (with 10 being “back to normal”) were used in the first 4 feedback questionnaires and in the final follow-up a global rating was also requested. During review appointments they had been frequently asked for a global rating following their description of function considering social connection, physical activity, education/work participation, symptom presence and recovery after any activity. The young person's rating was compared with concurrent physician and parental ratings based on their descriptions. A scale was developed based on their descriptions and rating (Appendix 3). A comparison between the distribution of ratings on the Bell and Global scale was conducted.

#### Comparison of Functional Rating and Reported Recovery

As additional data had been obtained from follow up questionnaires documenting the progress of the illness and providing feedback from those who had completed baseline information (*n* = 418), consistency in reporting outcomes could be checked.

The functional rating for both those who reported recovery and those who did not, was plotted to identify any overlap.

### Comparison of Reported Recovery and Functional Outcomes Between Two Groups

The demographics, proportion followed up, functional outcomes and reported duration of illness for both groups were compared to identify whether a systematic bias was introduced when young people return or fail to return baseline information. Returning information may reflect level of engagement, severity of illness or exercising choice.

### Association Between Questionnaire-Identified Baseline Depression, Anxiety, and Clinical Features With Outcomes

Mean baseline depression and anxiety scores were compared between those who reported recovery and those who did not using student's *t*-test. Presence of questionnaire-identified depression and anxiety were also compared with reported recovery outcomes. The presence of antinuclear antibody titers and outcomes were investigated using Chi square test. Statistica 13 (Statsoft –TIBCO) was used for all analyses.

### Feedback Regarding Management

Descriptive data regarding useful management, helpful information, helpful professionals, use of alternative therapies, and ways to improve management were collated and categorized.

## Results

### Baseline Demographics, Medical and Psychological Characteristics of Cohort

#### Demographics

A total of 784 young people with mean age of onset of ME/CFS 14.8 (range 6–18) years and M:F ratio of 1:3, were diagnosed with ME/CFS. The mean duration of illness prior to being diagnosed was 13.6 months (range 4 months−7 years). Socioeconomic status reflected the population based on Socioeconomic Index for Areas (SEIFA) data compared with census data across two census periods (18, 34). The rural/urban mix was proportionate to the population of Victoria however the ethnic mix was neither representative of the population of the state nor of the clientele of the hospital. Despite Victoria having successive immigration waves from over 200 countries, 80% of CFS patients had an Anglo-Celtic background (currently approximately 25% of the population), but predominantly Scottish/Irish descent, and another 11% were of northern European descent (Dutch and Scandinavian which is <0.5% of the population). Middle Eastern, African and young people of Asian descent were significantly underrepresented.

#### History Information

From 489 baseline history and psychological questionnaires distributed, 418 (85.5%) provided baseline history information. Fifty five were also given concurrent DePaul Pediatric questionnaires (35), of which 35 were returned (63.6%).

##### Onset

Eighty percent reported a defined onset following an infection, (most commonly EBV in 40% of cases), but a variety of other infections were documented such as CMV (10%), Mycoplasma, Toxoplasmosis, Varicella, Rubella, Parvo virus, Salmonella, Ross River virus, with 25% having documented serological change at the onset. Gastroenteritis and respiratory infections were commonly reported. Surgery for tonsillectomy or appendectomy in 2% and vaccination in 0.9% was identified as associated at the onset. Occasionally ME/CFS was diagnosed after a neurological insult or following a trivial infection in athletes who were overtraining (1–2%). Gradual onset was more commonly associated with orthostatic intolerance in young people with hyperflexible joints.

The peak onset was during winter with lower frequency at the beginning and end of the school year during summer and spring. Only 59% of the cohort (*n* = 683) had evidence of previous exposure to EBV and 35% of those tested (*n* = 600) had positive IgG for CMV. There was no difference in the prevalence of positive serology for either CMV (33% cf 36%) or EBV (58% cf 59%) between the groups with and without documented baseline information.

##### Symptom pattern

**Figure 1:**
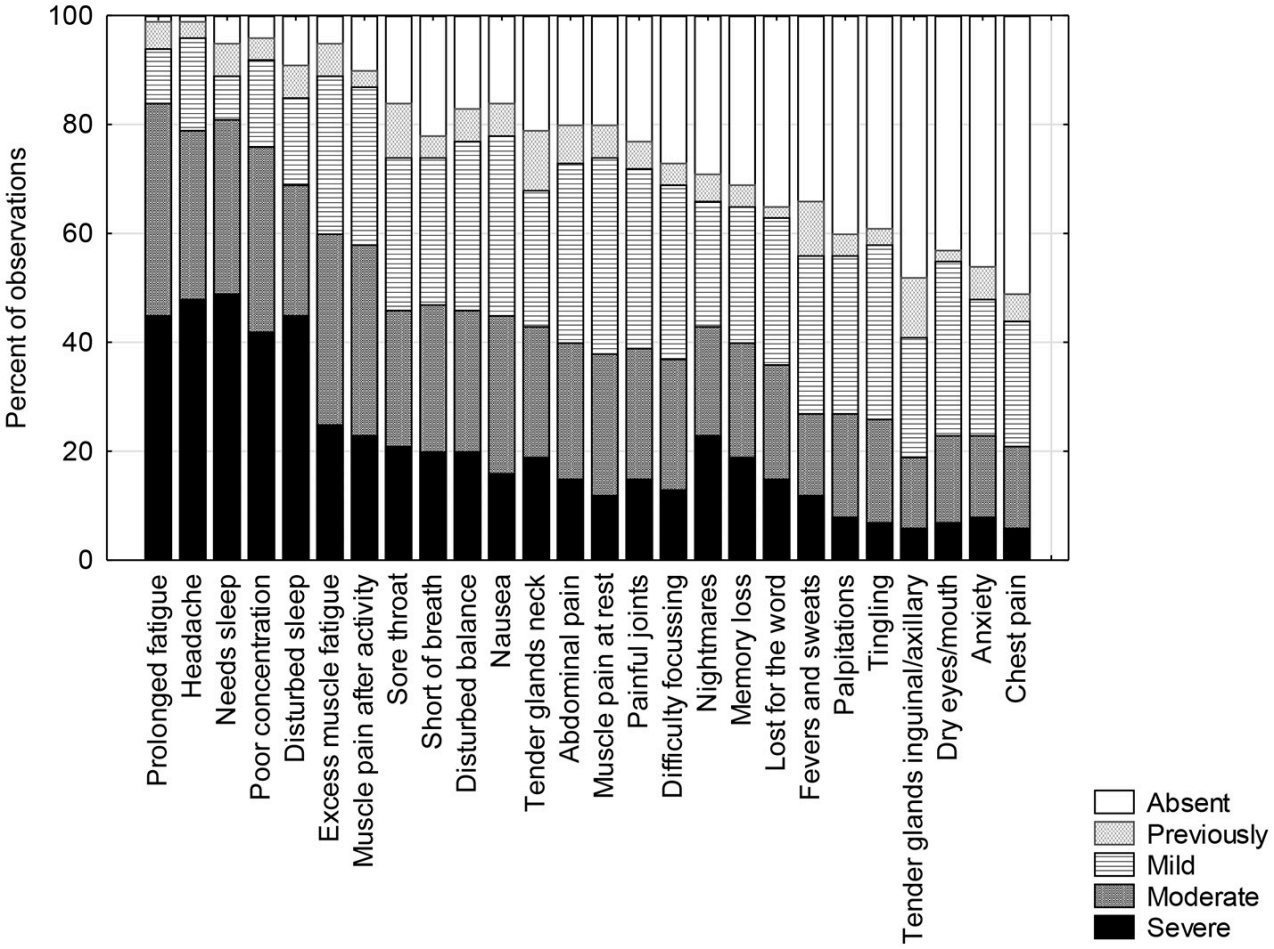
Frequency and severity of symptoms in young people with ME/CFS (*n* = 418).

**Figure 2:**
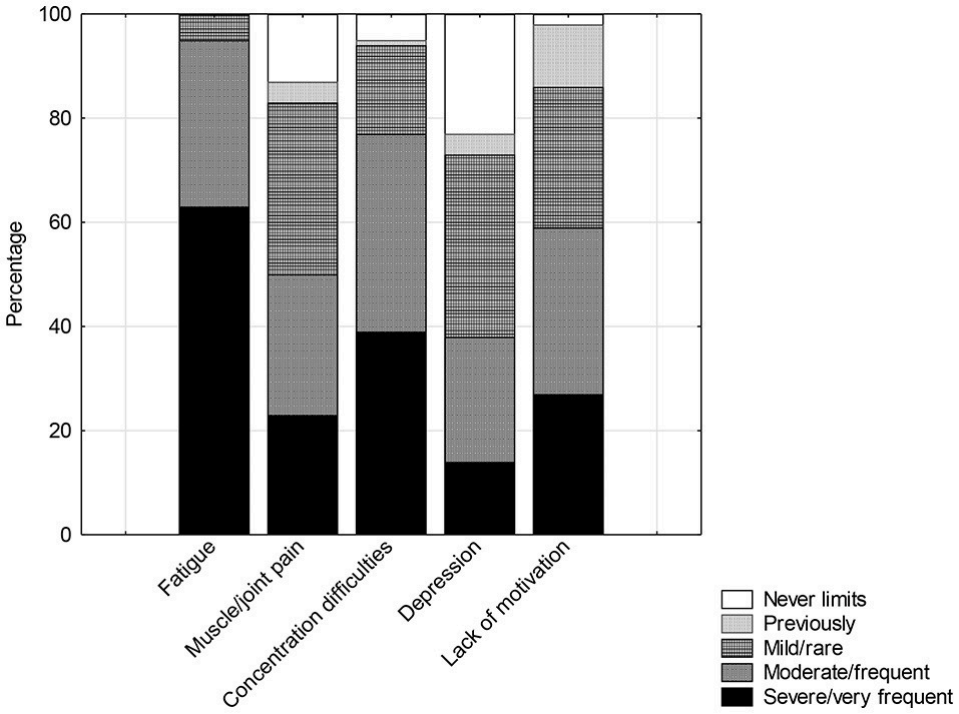
Rating of symptoms limiting functioning.

Symptoms of prolonged fatigue, persistent headache, needing excessive amounts of sleep, poor concentration, disturbed sleep, excessive muscle pain and fatigue after activity were reported in 90–100% of cases. Other pain, cognitive, “immunological symptoms” like sore throats, sore glands and felling hot and cold were reported by 60–80% of young people. Likely orthostatic symptoms, such as nausea, disturbed balance, difficulty focusing, tingling, anxiety and chest pain were reported frequently (more than 50%) also. The remaining 12 symptoms were reported in low frequency and also are not generally associated with ME/CFS (Figure 1). The most common 8 symptoms were reported with the same frequency and almost identical severity compared with the earlier separate sample (22). The remaining symptoms were comparable and again significantly different from the community sample of age and gender matched adolescents. Fatigue, concentration difficulties, motivation and pain were identified as limiting activity (Figure 2) whereas depression was less so.

Fifty eight per cent reported a continuous pattern of illness with fluctuating severity, 14% continuous at same level of severity, 12% relapsing and remitting pattern, 9.5% relapsing in past now continuous and 6.5% continuous in past and now relapsing.

##### Baseline depression (Beck), anxiety (Spielberger) and General Health Questionnaires

**Figure 3:**
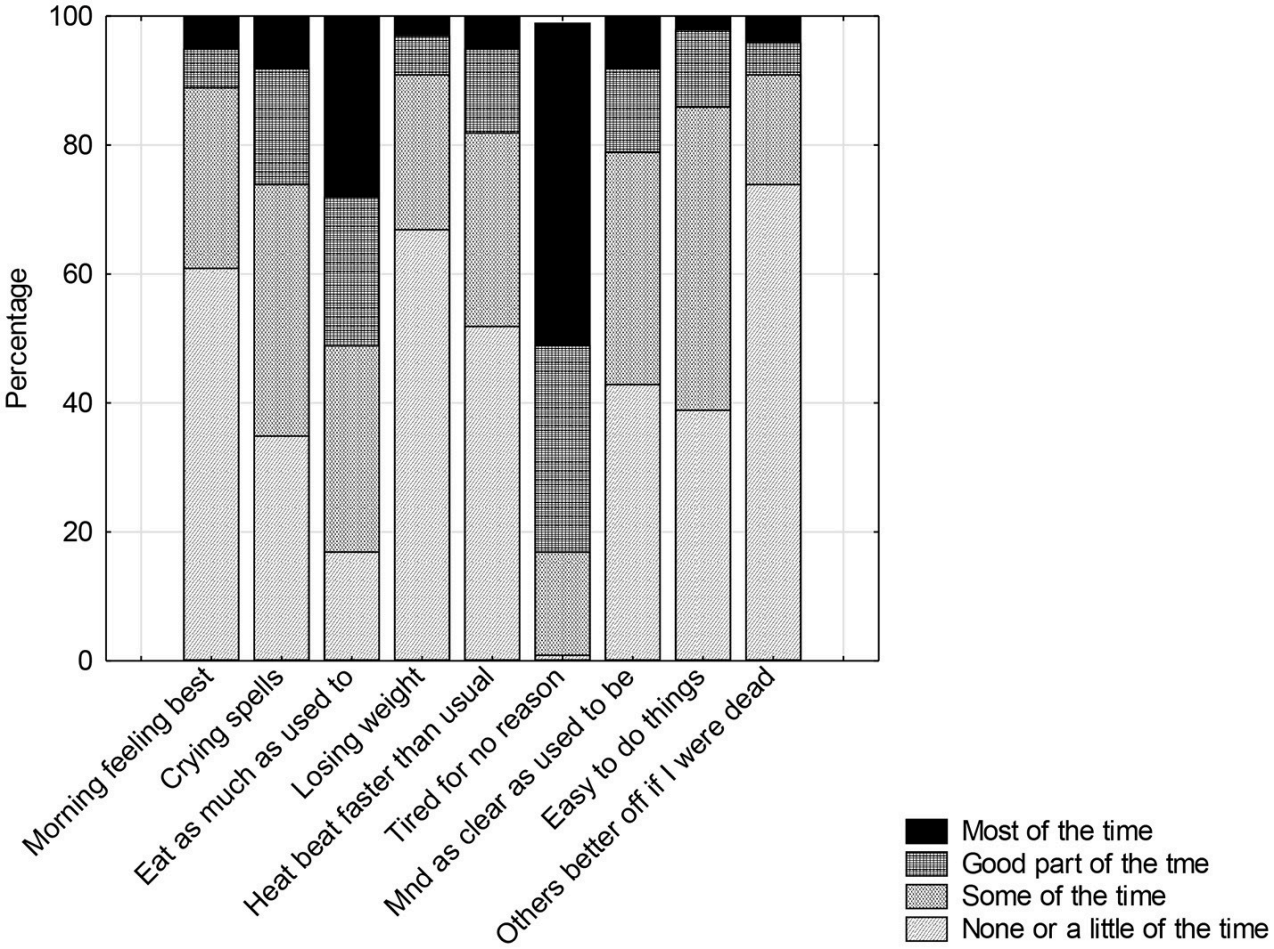
Rating of frequency of specific symptoms in ME/CFS.

The mean total score on the Beck was 13.8 sd 8.9 (range 1–51) (*n* = 370). On this scale, scores between 0 and 10 indicate “normal ups and downs” experienced by 45%, 10–20, “mild mood changes” (35%), 20–30, moderate depression (15%), 30–40, significant depression (4%) and >40 severe (1%). Items related to “ability to work,” “tiredness,” “sleep disturbance,” “ability to make decisions,” and “feeling dissatisfied” scored highly (mean for each item 1.3–1.7, range 0–3), and as they are associated with CFS, the scores may as a consequence be inflated. Twenty per cent of the cohort scored more than 20 while 5% were in the moderately severe range. Less than 1% reported anhedonia symptoms (feeling “hopeless,” “bad or worthless,” “disgusted with themselves,” or “feeling as if they deserved to be punished”). Direct questions regarding aspects of depression, motivation, concentration and effects on functioning revealed a consistency in responses within the baseline questionnaire (Figure 2), It was noted that 25%, at least “some of the time” (Figure 3) thought that their family “would be better off if they were dead” however less than half these were moderately depressed based on the Beck where 1% reported suicidal thoughts. Community surveys of adolescents in Victoria have noted 18% (CI 17–20) of young people report significant depressive symptoms (36). Thus, the rates of depression in this clinical sample were reported at only marginally higher rate than the adolescent population. Higher scores were associated with severity of symptoms, not feeling supported by family, the medical profession or school. The mean score, however, was significantly different from a small community sample of age and gender matched young people (Table 1).

**Table 1:** Comparison scores for Depression (Beck), Anxiety (Speilberger), and General Health Questionnaire (GHQ) for study group compared with previous clinical and community age and gender matched samples.

	**Current clinical**			**Previous clinical^a^**			**Community sample^a^**				
	**n**	**mean**	**sd**	**n**	**mean**	**sd**	**n**	**mean**	**sd**	***t*-value**	***P*-value**
Beck	370	13.8	8.9	159	12.5	7.0	65	5.3	5.7	7.97	<0.0005
Spielberger	259	88.9	24.9	118	85.4	22.4	65	69.6	17.7	5.28	<0.0005
GHQ	277	16.0	3.01	171	16.6	7.0	66	10.1	5.3	7.8	<0.0005

a *Rowe and Rowe (22)*.

The mean “state” scores for the Spielberger was 43.9 sd 12.6 (range 21–79) (*n* = 273) and the mean score for the “trait” was 45.1 sd 12.8 (range 21–80) (*n* = 259). The mean total score was 88.9 sd 24.9 (range 40–157) which was higher than the previous clinical sample and significantly different from the community sample. Approximately 30% of the young people with ME/CFS experienced moderate levels of anxiety when diagnosed.

The mean General Health Questionnaire score of 16.0, sd 3.01 (range 9–27) was similar to the previous clinical group but significantly different from the community sample (Table 1).

##### Anti-nuclear antibody titers

Thirteen percent of the sample (*n* = 442) had a titer between 1:40 and 1:80, however the reporting level changed in 2008 from 1:40 to 1:160. Twenty one per cent of the sample (*n* = 499) had a significantly positive titer comprising 10% with 1:160 (mildly positive), 9% between 1:320 and 1:640 (moderately positive), and 2% between 1:1,280 and 1:2,560 (strongly positive). The described patterns were speckled (65.5%), homogeneous (3.2%), nucleolar (15%) and mixed speckled/homogeneous (15.9%). The high titers were not associated with clinical signs or any other markers of connective tissue disorders such as high sedimentation rate, positive antibodies to double stranded DNA, or extractable nuclear antibodies, and this was confirmed by a rheumatologist. The proportion of positive titers is at least double the expected rate in this age group, and although it not unknown to have moderately strong positive titers without evidence of connective tissues disorders in pediatric rheumatology clinics, the proportion of moderate to strongly positive titers is much greater than expected (personal communication-J Akikusa, Royal Children's Hospital, Aug 2018).

### Follow Up Study

#### Duration of Illness

**Figure 4:**
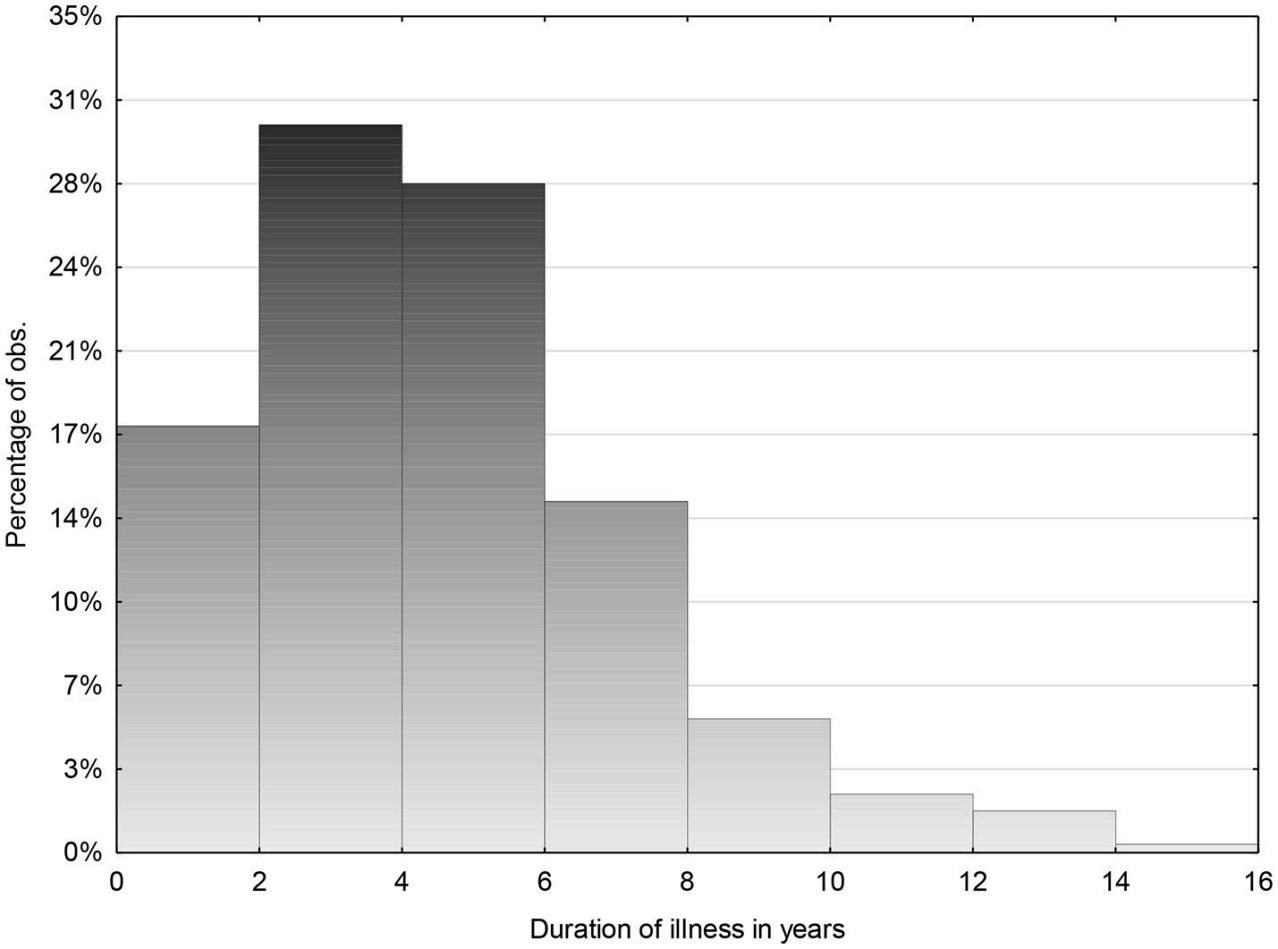
Mean duration of illness for total group 5 years (*n* = 298) sd 2.7 (1–15).

From the total group of 784 (M:F ratio of 1:3) and mean age 22.5 years sd 4.6 (range 7–35.7) data were obtained from 81.8%. The mean duration of follow up was 8 years sd 4.3 (range 1–21 years). Mean duration of illness for total group was 5 years (*n* = 298) sd 2.7 (range 1–15) (Figure 4) and 47% reported recovery.

For those followed for <5 years 29.8% (55 of 185) reported recovery and for those followed for longer than 5 years, 127 of 456 (27.9%) reported recovery occurring before 5 years. However, for those followed for between 5 and 10 years, 54% reported recovery (233/431), and for those followed for more than 10 years 68% reported recovery.

#### Comparison Between Groups With and Without Baseline Information

From the 418 young people providing baseline information, 364 (87.1% return rate) provided formal follow up data on a total of 971, mean 2.5 (range 1–7) occasions. One third (*n* = 122) provided one response and the remainder (*n* = 242) provided a mean 3.5 (range 2–7) responses a minimum of 2 years apart. This enabled regular feedback concerning management and documentation of any relapses and confirmation of duration of illness close to the occasion rather than relying on memory.

From the remaining 366, 277 (75.6%) responses were obtained, and 30 provided formal follow up information on more than 1 occasion. The group without baseline information contained more young people who were still at secondary school, thus reflecting the difference in age distribution and use of services (*t*-test for independent samples *t* = −3.33 df 508 *p* < 0.001 F-ratio variances 1.68 *p* < 0.00005). There was also a significant difference in mean duration of follow up 7.6 years sd 4.5 (range 1–21.6) years compared with 8.3 (1–19) years (*p* < 0.05) and the proportion reporting recovery was significantly different 40% compared with 52% (Chi-square 6.8, *p* < 0.01).

As the majority of comparisons including: duration of illness (4.9 cf 5.1 years), functional score if reporting recovery (9.0 cf 8.8), duration of illness until help received (13.4 cf 13.8 months), proportion working or studying full time (61% cf 63%), proportion caring for children (5%) or not working or studying (5%) were not significantly different, the general comments and observations have been combined from the two groups (see Table 2).

**Table 2:** Comparison data between study and comparison groups.

Total **cohort *n* = 784 (**M:F 1:3) Mean **age 22.5** years (range 7–35.7 years) Follow up (**FU**) data from **641** (**81.8%**) Mean **length FU 8** (1–21) years Proportion reporting **recovery 46.5**% Mean **duration illness 5 years (1–15)** (***n*** = 298)		
	**Study group who provided baseline data *n* = 418**	**Comparison group *n* = 366**
N providing FU data (%)	**364** (87.1%)	**277** (75.6%)
M:F	23%:77%	27%:73%
Age at FU (range) years	23.2 (14.6–33)	21.9 (7–35.7)^**^
Mean length FU years	8.3 (1–19)	7.6 (1–21.6)^*^
Mean duration illness (months) until help/diagnosis	13.4 (3–72)	13.8 (3–84) (ns)
Proportion reporting recovery	52% (*n* = 188)	40% (*n* = 110)#
Mean duration illness (years)	4.9 (1–14)	5.1 (1–15) (ns)
Mean functional score (if recovered)	9.0 (5–10)	8.8 (5–10) (ns)
Working/ studying full time	61%	63% (ns)
Using/used Visiting Teacher Service	20%	20%
Used Distance Education	11%	24%
Completed or undertaking post-secondary education	69%	62%
Not working/studying	5%	5%
Caring for children	5%	5%
Receiving Government Disability Support	26%	33%

* t-test p < 0.05

** *t-test p < 0.001. #chi square 6.8 p < 0.01. Bolding indicates composition and outcomes for total cohort*.

#### Educational Outcomes

Only 5% of those followed up reported not working or studying, with 8% working or studying less than half time, 24% more than half time and 63% reported working or studying full time. Some of those reportedly not working or studying were traveling overseas prior to university whereas others remained very unwell or had other illnesses. Similarly, 62% reported that they were currently studying at either a secondary or tertiary level. Of the 66% who had undertaken a post-secondary course (tertiary education) half were still studying. Twenty per cent were also working part time while they were studying. Twenty per cent reported using the Visiting Teacher Service during their secondary education and 16.5% used Distance Education service either solely or supplementing their school attendance. Five per cent reported having children, some of whom were working outside of home as well.

For comparison, the 2011 census (18), indicated that 75% of 15–25 year olds complete secondary education and 60% undertake post-secondary education, with 6% unemployed, 9% not in the workforce and 26% in employment. The population census reported 85% of 15–25 year olds are fully engaged in either work or study or both.

A wide range of courses were undertaken by the cohort ranging from traditional University courses to ones with high entry criteria such as medicine, law and health sciences. Technical courses, diploma courses and some apprenticeships were also reported. Several had also completed Masters degrees and Ph.Ds. A very wide variety of occupations were reported from aeronautical engineering, health sciences, law, trades and with some working in the service industries. The majority of service industry jobs were part time for support while undertaking study. A very small percentage did not complete secondary education and this was reported associated with other illness, social circumstances or an unsupportive school.

Disability support from the government was received by 29% of the group. The majority was studying while receiving this or working part time. At the time, government policy allowed this support to enable part-time completion of a degree for those who were chronically unwell, without having to work as well. Of the 5% not studying or working outside of home, several were mothers, one was a carer for a parent, and others had an additional illness or were doing voluntary work.

#### ME/CFS Functional Score and Reported Recovery

As some young people found the concept of recovery difficult, they were asked to describe how they thought they were functioning with a rating of 10 being “very well” or “back to normal” and 1 being “bedridden.” They were also asked what they meant by their score and it usually included a sense of the amount of activity, work or study that they were able to manage, what their stamina was like, how well they recovered from any activity, as well as social engagement and presence of symptoms. The young people were remarkably consistent in what they included in the scoring and how they described it. The clinician also rated the young person based on their reports and found high inter rater reliability for the scores (90%). Parents were generally also asked and they agreed in 80% cases. They often rated one point below the young person as they tended to compare them with how they “used to be.” The descriptors of the global rating are in Appendix 3. Young people reported that the central part of the Bell CFIDS Scale was difficult to score and they preferred to use the global rating (ME/CFS Functional rating). Concurrent data were collected to allow comparison and this difference in scoring in the mid range (between 4 and 8) was confirmed. The correlation was 0.833 (*n* = 252), however the correlation between the two scales was less in the mid-range (0.65) with the ME/CFS Functional Rating tending to score higher. The Bell scale had an irregular distribution with scores 9 and 10 scoring with higher frequency but low frequencies in the mid-range and an increase at the lower end.

The study group was asked to assess their poorest level of functioning using the Bell scale and 98.7% scored <5 while 68% scored 2 or less. They were also asked their current level of functioning using the same scale as well as the functional rating.

**Figure 5:**
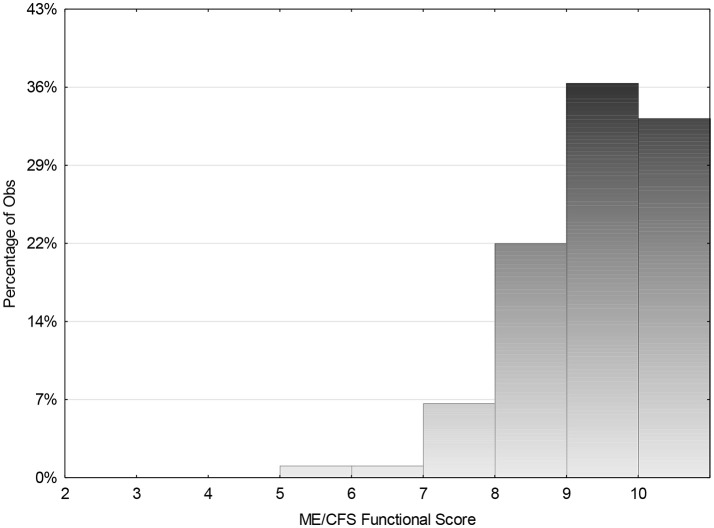
Follow up global functioning score for those who indicated that they had recovered from ME/CFS for cohort (*n* = 298). Mean score 8.9 (range 5–10).

**Figure 6:**
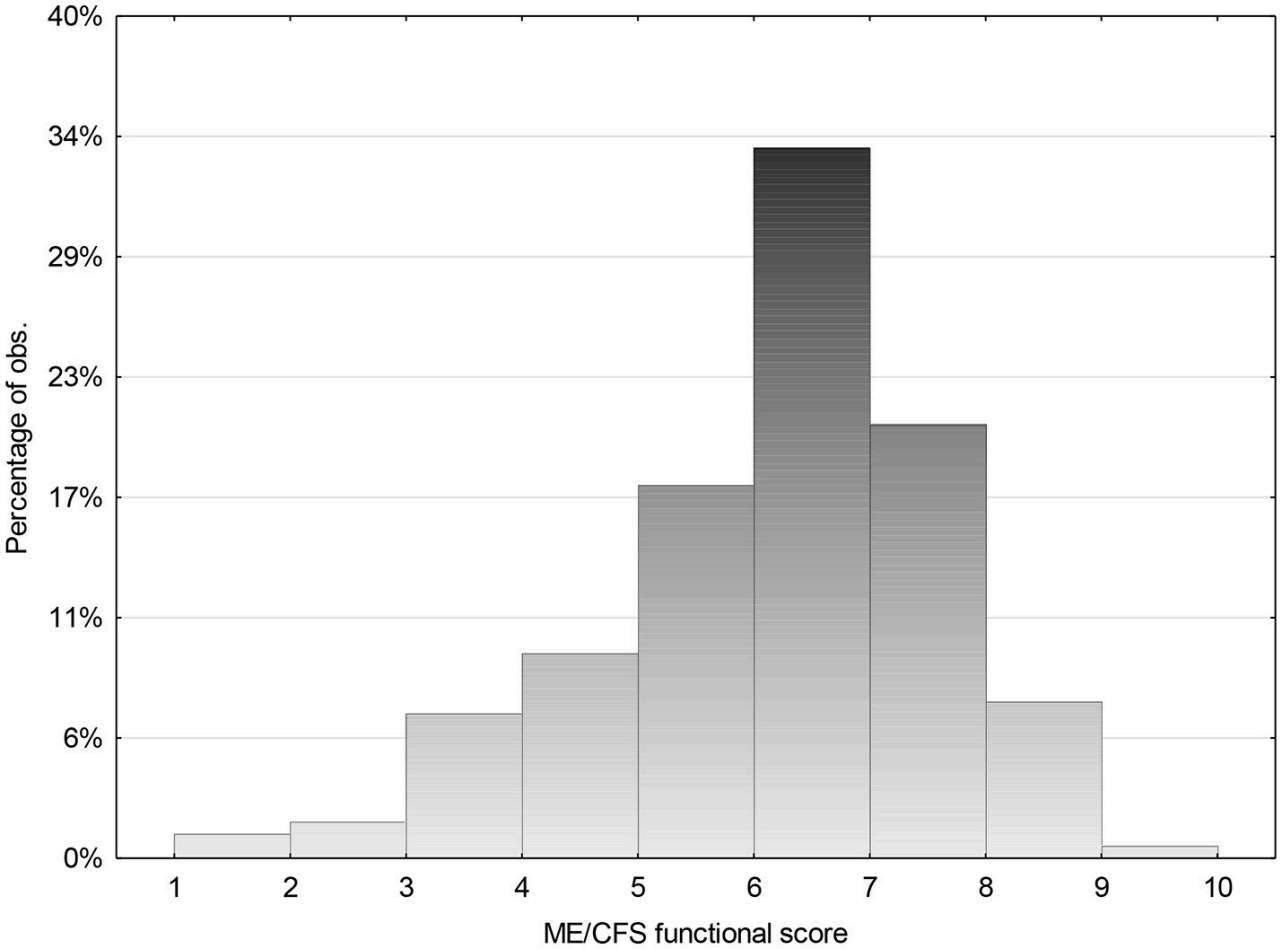
ME/CFS Global functional score for those who reported that they had not recovered.

The range of ME/CFS Functional score for those who reported that they had recovered was compared with those who reported they had not recovered. (Figures 5 and 6) There was a significant overlap. Some scored low due to other illnesses that they differentiated from CFS. Others reported that they did not know what was “normal” as it had been so long since they were well. They reported that “how a 22 year old spent their energy was different from 15 year, but they were not sure what that should be.” Others, who reported themselves as “well,” felt that they were managing well, but their parents made the comment that they “did not think that they had the stamina that they had demonstrated as an adolescent.” Some needed to be “perfect” to describe themselves as “well” whereas others compared themselves with how they were when they were first unwell and were very grateful to be able to do what they were currently able to do. Some who felt they had recovered also scored lower, as they were caring for small children, were working part time and felt sleep deprived. Others admitted that they were dealing with depression as an additional issue. Hence many factors influenced whether recovery was reported, as well as whether the level of functioning was solely related to ME/CFS.

#### Reports of Recurrent Infections and Prolonged Recovery Afterwards

The majority reported a reduction in the number of infections and the fact that they rarely had upper respiratory infections when the rest of the family was unwell. They did notice that when they were starting to feel better they commonly had an increased number of upper respiratory infections over a year or two which then settled to a normal rate. This did trigger significant panic and low mood as the symptoms they experienced with these common illnesses reminded them of the early days of ME/CFS and they felt that they could not deal with prolonged illness again. As these illnesses commonly recurred on return to school they acknowledged that it made them more cautious about engaging again. They also clearly did not believe reassurance that feedback from others who had experienced similar events, indicated that recovery is generally short lived. Comments on feedback forms indicated the fear and anxiety was excessive but not warranted. Those who had had regular asthma prior to becoming ill, observed that they rarely had asthma when they were very unwell with ME/CFS. Approximately 5% had documented EBV infection when still unwell but only one young person who had significantly improved at that stage, had a prolonged recovery of several years. The remainder reverted to their usually level of functioning in <3 months.

#### Other Illnesses

The majority of associated illnesses reported were depression (rarely associated with depression at baseline), severe anxiety, panic disorder, and POTS and neurally mediated hypotension. There were individuals with agoraphobia, eating disorder, irritable bowel disease, celiac disease, Graves disease, Rheumatoid arthritis, and several developed Systemic Lupus Erythematosis (SLE) but all but one of these had negative ANA at the onset of CFS. Two struggled with drug addiction. One young woman had died at 22 from neuroendocrine carcinoma of the cervix and another had breast cancer at 23 who incidentally reported having a reduction in her CFS symptoms after chemotherapy. At least 10 young women disclosed sexual abuse years later, the majority of which occurred after they were unwell, thus adding to the trauma.

#### Members of Family With History of ME/ CFS

Although data from the baseline questionnaires indicated 17% had a close family member who had a prolonged recovery from an infection lasting more than 6 months, a diagnosis of CFS in the past or currently, only 12% of that group reported that a family member has had, or has ME/CFS. In contrast, 15% of the remaining cohort reported a family member with a history of ME/CFS. Parents, siblings and cousins were identified, with some families having three immediate family members with the diagnosis.

### Any Predictors of Outcomes?

#### Presence of Positive Antinuclear Antibody Titers

The proportion of positive titers (>1:160) was at least double the expected rate in this age group (37, 38), and it is expected that titers increase with age. However, subsequently only 4 (0.5%) patients had diagnosed SLE. Of these, 2 had titers of 1:160 (speckled pattern) and one had a titer of 1:1,280 homogeneous pattern at age 15, but no other features. One with later rheumatoid arthritis and 2 with Sjogren's disease had negative ANA titers, while one who developed Grave's disease had a titer of 1:320 with speckled pattern.

There was no significant difference in reported recovery rate and level of ANA titer [General Linear model *F*_(1, 76)_ = 0.12 *p* = 0.73]. Those with high titers (1,280–2,560) reported recovery from CFS. Of those with a titer of 1:640 or higher, there were no follow up data available for 25% of them. Of the remainder, in ¼ the titer remained stable, ½ the titer reduced to normal or near normal, and ¼ the titer increased without any other clinical features of disease. Therefore, high titers were not associated with “non-recovery” and there was a suggestion that titers reduced with recovery.

#### Outcomes for Severe Symptoms at Outset

The rating for level of ill health early in the illness was a mean score of 1.9, sd 1.3 (range 0–5) with 98.7% scoring <5 and 68% 2 or less, using the Bell CFIDS score. At worst, the majority were bed ridden and unable to attend school at all, or at the most, attended for short periods during the week. Therefore, in this study the majority of young people had severe illness. Severity of illness was not predictive of “non-recovery.”

#### Relationship of Identified Depression, Anxiety, or GHQ Scores to Outcomes

There was no difference in depression score at baseline between those that reported improvement (mean 14.46) and those that had not (mean 13.17) df 315. *t* = −1.3 *p* = 0.18. Although data were available on follow up from 86% of those with Beck data there was no significant difference in baseline score between the total group and those followed up (mean_1_ = 13.85, mean_2_ = 13.78; df 688 *p* = 0.91). If selected for higher depression scores at baseline (20% of sample) there was no difference in baseline score between those that reported recovery and those that did not (means 28 and 27.5) i.e., depression at baseline was not predictive of outcome.

There is also no difference in anxiety score at baseline between those who reported recovery and those who did not, so anxiety at baseline was not predictive of outcome using student *t*-test (mean_1_ = 28.1, mean_2_ = 27.5; *t* = 0.4 *p* = 0.7).

Similarly, the GHQ score at baseline was not predictive of recovery (*t*-test −0.62, df 233, *p* = 0.5).

#### Predictors of Good Functional Outcome

There were no obvious predictors for outcome. However, as the young people reported how important remaining engaged in education and socially connected, as well as feeling supported, encouraged and being believed, and how difficult it could be to deal with social isolation, social confidence and unsatisfying work when still unwell, it could be inferred that improving these circumstances improves their ability to cope with their situation and function better. Use of disability support during post-secondary education was very important, if they were not able to work as well as study part time. Ensuring that they could continue study at a reduced rate without having to try and find work to support themselves was key. Frequently when they had completed their studies and able to find a job, even if part time, they were often in a much better position to support themselves and feel independent.

### Feedback Regarding Management

#### Feedback Regarding Helpful Professionals

Twenty three per cent of the group providing baseline information “did not find any professionals helpful,” however only 17% of the remainder did not find professionals helpful despite our concerns that this group may contain a higher proportion of young people who did not engage with health services. Pediatrician, Visiting Teachers, cardiologists who were consulted because of orthostatic intolerance were most frequently cited, and less frequently physiotherapist, family doctors, some school staff, counselors, psychologist, massage therapist, sleep physician and gastroenterologist. The reasons they were found helpful related to “being taken seriously,” “being believed,” “providing support,” “providing practical strategies,” “alleviating symptoms,” “providing educational liaison and advocacy,” “dietary advice,” and “managing other illnesses” that may also be present. The most common responses were appreciating “being understood,” “feeling respected,” “supported,” “reassured,” and “not feeling alone.”

#### Feedback Regarding Helpful Information

The young people consistently indicated that the most helpful information was providing some management strategies to help them “feel in control of their life again.” Managing symptoms and providing information regarding the importance of good routines particularly around sleeping and eating and activity was also appreciated, as well as understanding how to monitor their activities. Strategies for re-engaging with peers, assistance with liaising with schools as well as providing information regarding options to remain engaged in education were rated highly. General information about the illness prognosis and what had helped others, as well as up to date research information was useful. In addition to the hospital clinic, information was sourced from the internet, ME/CFS Society newsletters, and the support groups.

#### Could the Illness Have Been Handled Better?

There were some differences in feedback from the early questionnaires compared with the later ones mostly related to lack of awareness of the illness. They indicated the need for an earlier diagnosis, and more understanding by the community, the medical profession and by schools. They reported how important it is “to be believed” and to find “someone who understands.” There were regular comments about the perceived arrogance of the medical profession and lack of understanding, and how distressing it was to be accused of “being lazy” or implying that it was “all in the mind.” Lack of understanding and flexibility by educational institutions was reported as a major source of distress.

In later follow up returns, the comments regarding the need for general understanding of the illness and their plight, the need for earlier diagnosis and access to management strategies were similar. There were comments regarding self-management that they thought they could have managed better, such as managing stress, routines, pacing, depression, exercise and being more open to acknowledging the presence of depression especially as they were improving and re-engaging in society. There were comments regarding the need for resources to be available for the family, as well as regular but not necessarily frequent follow up appointments, and that contact with other young people in a similar situation would be helpful. Many commented that they also needed to be willing to ask for help.

Many indicated the need to be sensitive about when psychological assistance is offered. They were often not ready for that intervention as they felt they had enough to cope with. They were sensitive as to whether this was implying that psychological issues were the “cause.” Many indicated that they were “miserable” and “fed-up with being unwell” but not depressed which on reflection was a healthy response to their situation. If they were happy and content with not being able to attend school, see friends, or do any physical activity it would be a cause for concern. Others reported that they struggled with motivation and seeing any end to the illness. Some had a family history of depression and were open to receiving assistance. It was noted that when many young people started to feel a little better they were willing to have strategies for managing stress and anxiety and also reassurance that they had developed resilience while learning to manage the illness, and if faced with challenges in the future would have the skills to cope. Some young people needed help with family issues. Others needed help with integrating back into school and dealing with the challenges of social interactions and social learning tasks of adolescence, particularly as they felt vulnerable and had lost some confidence.

#### Use of Alternative Therapies

Seventy per cent of the young people used alternative therapies and these included 40 different types, some trying up to 10 different ones. The therapies ranged from naturopathy, chiropractics, homeopathy, Chinese herbs, intravenous vitamins, Reiki, Qi Gong, Tai Chi, Yoga, Myotherapy, Bowen therapy, massage, hypnosis, cupping, aromatherapy, color therapy, meditation etc. There were very few therapies that were considered of any value by the young people. The only ones that approached a 30% positive response involved massage (under a variety of different guises) for those with muscle pain and “good dietary advice” often in young people with associated abdominal discomfort. The most common comment was that they “wished their parents had not wasted their money or their energy by taking them to people who had promised to cure them but didn't.”

## Discussion

### Demographics and Baseline Symptoms

The finding of M:F ratio of 1:3 is consistent with other studies showing a consistently higher proportion of females to males with CFS, for example, Reyes et al. (39), Rowe et al. (40), and Bell et al. (7). The observation in this study that the ethnic background characteristics are not consistent with the demographics of the state, nor the clientele of other general medical or adolescent health clinics in the hospital, is interesting in the light of reports of CFS occurring in community surveys in Japan (41), Korea (42), and the United Kingdom (43). However, each of these studies was in adults and associated with high rates of psychological distress and based on survey information rather than a clinical assessment of CFS. There may be ethnic and cultural differences in how CFS is reported, for example in Japan it is rare for infection to be recognized as the trigger, and chronic sleep deprivation and stress are noted to be associated with the condition, where it is commonly described as school phobia (personal communication, Prof. Miike, 2008). Shi et al. (44) reported likely CFS from surveys of Chinese students associated with despondency and anxiety regarding school. There was a predominance of males. There are few documented pediatric studies in other ethnic populations. Due to the universal health care system and the demographic of other medical clinics in the hospital, access to health care is unlikely to be the main explanation. This cohort reported a high rate of post infective onset, as well as higher than expected rates of positive antinuclear antibody serology without evidence of clinical connective tissue diseases. It is recognized that people of northern European descent have a higher proportion of autoimmune diseases both in ethnic variation in expression of autoimmune diseases such as SLE (45) and the prevalence of multiple sclerosis (46).

Of note, was the consistency with which this cohort of young people responded regarding the severity and frequency of symptoms that was comparable to the earlier cohort that was obtained when there was very little awareness of the condition in young people (47). The high rate of reported infections at the onset and the consistency in responses suggests a relatively homogeneous group. The rates of anxiety and depression were also comparable with the previous sample and higher than the community sample. It is noted that the baseline rate of reported depression was only marginally higher than a large concurrent adolescent survey in the state (36). Crawley et al. (48) and a review by Lievesley et al. (49) have reported increased prevalence of anxiety and depression in young people with CFS implying that this association may be contributing to the morbidity.

### Follow Up Study

### Outcomes

Follow up of this large well defined cohort with 87% of the study group providing data, has indicated that more than half the young people report recovery and many who do not report recovery, are also functioning well. The mean duration of illness was 5 years (range 1–15 years). By 10 years 68% reported recovery. The young people who reported recovery at 14 and 15 years could not identify any contributing factor. “It just happened.” The duration of illness was comparable to the placebo arm in the 5-year follow up following the immunoglobulin trial and a mean of 8 years from onset (13). Sixteen percent were moderately unwell with 20% reporting “not quite back to normal” and the remaining 64% reporting recovery. Bell et al. (7) had a well characterized group also with a defined onset and likely post-infective, that was followed for approximately 13 years. Their findings were similar in that all had improved function and most considered that they had recovered except 20% remained unwell. Other follow up studies have been for short periods (1, 4, 6, 50); have not been diagnosed clinically (2); had half the cohort with fatigue of 1–6 months duration (4); the cohort was small in numbers (3); or the follow up did not have a high proportion participating (4, 5). Due to these differences, findings are difficult to compare. In this study, it is clear that there was variation in how young people defined recovery. There was a significant overlap in functional scores between those who had reported recovery and those that had not. In this study, as well as requesting an assessment as to whether they have recovered, an indication of their level of functioning that took into account stamina, recovery after activity, proportion of participation in work or school, whether the workload has been reduced, level of social contact and severity of symptoms. Early follow up questionnaires requested this in detail as well as using the Bell scale. Young people could consistently estimate their level of functioning. A global functional rating scale (Appendix 3) compiled from the surprisingly consistent descriptors of hundreds of young people with ME/CFS in the clinic has good inter-rater reliability. This however did not necessarily reflect their concept of whether they had recovered or not. They reported struggling with understanding what to expect as “normal functioning” as they had no frame of reference.

Other follow up studies have varied in how they have reported recovery making comparisons difficult. Differing measures such as measures of fatigue (8), symptom presence and functional outcomes, (9, 10), self-report (6) or a combination of global functioning and self-report have been used (7). Parslow et al. (11) have been investigating what aspects for defining recovery are important to young people with ME/CFS. Fatigue scales, Karnovsky scores, school attendance or physician's perspective (51) are useful markers of improvement but do not appear to capture the complexity of what is important to young people as well their tolerance level to the normal “ups and downs” of life and their satisfaction with what they are able to do.

In this follow up study there is a higher than expected rate of engagement in post-secondary education compared with the community rates, and a high proportion (95%) who are either working or studying part or full time. It was also noted that those who attended tertiary education, there was a much higher than community rate of completion of courses and lower than community rate of changing courses. In Victoria, there is a scheme available to apply for Special Entry Access to university if preparation for university entrance had been impacted by chronic illness, and usually very supportive Disability Liaison Units exist in universities to assist once enrolled. Universities recognize that young people who have faced adversity do not tend to waste their opportunities and their applications are usually given consideration.

It is noted that the proportion of young people with positive ANA is higher than expected without progressing to clinical autoimmune disease. There was a range of other illnesses reported which influenced the functional rating but were usually reported as separate from ME/CFS. Severe anxiety, depression, agoraphobia, eating disorder, although uncommon was linked with ongoing CFS. Presence of reported depression at follow up was linked with family discord or lack of support, difficulty with completing an education to equip for satisfying work, ongoing severe symptoms or disclosed history of sexual abuse that had often occurred after the onset of the illness.

### Feedback

Regular feedback has helped inform our management and advice that we can offer young people. There has been a clear message about how important it is to the young person to be believed, to feel supported and to be provided with a management plan that enables them to have some control of their lives. Maintaining social connectedness and support for continuing education is crucial to ensure their aspirations remain. Similar to the reported feedback from young people in the UK, advocacy for school and ability to continue with education was valued (11). The education system in the state allowed for flexibility in workload, school attendance, mix of Distance Education and school based learning as well as access to a Visiting Teacher service whose brief is to help liaise and coordinate the learning program at school and at home until the young person is well enough to return to school full time. Appropriate documentation to access these services is required but experienced Visiting Teachers explaining to other teachers significantly improved cooperation and satisfaction on the part of student and staff.

Anecdotal information about the perceived value of various alternative therapies has been appreciated by young people. As few therapies have even reached expected level for placebo intervention, the time and money saved has been often been used to support an interest area that has contributed to enriching their life.

Feedback indicated the importance of being sensitive about when psychological assistance is offered as there were many situations where they were not receptive until later in the illness. Sometimes this was related to an implied attitude that ME/CFS is a “psychological illness” rather than a chronic illness with its attendant challenges.

If graded exercise was implied as a means to recovery and symptoms were made worse, the young person often withdrew from any medical assistance and frequently from school. Some interpreted the message as indicating that the problem was with their cooperation or motivation, not with what was being advocated. Several were very angry and suspicious of medical profession. At other times, intervention programs were very helpful when self-management strategies such as organizational skills, sleep hygiene, advocacy for appropriate school program and appropriate strengthening exercises and advice regarding how to monitor increases in activity including “boom/bust planning.”

Many reported that they appreciated the plan that recognized the impact of illness on every aspect of their life and the fact that they could reduce this effect by not neglecting these areas. This approach was not disregarding the fact that they were ill or implying that their belief that they were unwell was hampering their recovery. If they felt they were believed, they did not have to prove it, and they just needed to find a way to survive it in good shape and feel that they were supported in doing so. Families certainly indicated that they were grateful and much less anxious when there was a negotiated plan and they were supported in dealing with the education authorities. Similarly, the education authorities reported being grateful for information and support.

Although a variety of management strategies such as adaptive pacing, graded exercise, cognitive behavior therapy can be helpful (14), the specifics of what this entails and whether it is implemented as an individualized program is often not clear. However, Burgess et al. (52) found a family focused individualized home based rehabilitation program was well received for those with severe CFS. Evidence for significant improvement is hampered by difficulties in comparing outcome measures according to clinical presentation, patient characteristics, case criteria and degree of disability (16). The routine care that occurred in this cohort does include aspects of these strategies but as no two management plans were the same, the details of how they were implemented were not standardized. What did seem important was that the young person was devising the plan with the support of the family and cooperation of the school.

### Outcomes and Baseline Characteristics

Although many reports (3, 43, 49, 53, 54) imply that the presence of depression or anxiety at the onset could potentially influence the recovery or function, there was no convincing evidence in this study that the presence or absence of depression, anxiety or illness severity was associated with outcomes in this cohort. Neither the presence of positive ANA titer nor documented EBV infection was associated with outcomes. Nonetheless, depression, anxiety or other illness documented at follow up was reported as affecting function and occasionally was linked with ongoing CFS or else reported as a separate issue following recovery. Therefore, this study was not able to identify any predictor for recovery.

### Strengths of This Study

The desire by young people to have answers to common questions ensured a high participation rate and frank responses from the large cohort over a long period of time. There was a very high proportion of the original cohort who willingly provided information despite the challenges in tracking such a mobile population. Despite little information in the public domain about the illness when some of the initial symptom inventory and background information data were collected, the consistency of responses indicates that the group is relatively homogeneous and consistent with earlier and later case definitions (19, 20, 35, 55) as well as being comparable to an earlier study group (12, 22). Although the comparison group in this cohort did not have comparable detailed baseline data nor the prospective repeated follow up questionnaires the 2 groups were only significantly different on age range and current education status but all other comparisons were comparable. Similarly, the concern that young people with severe CFS may not be represented in this study and therefore skew the findings, was not realized as some attended in wheel chairs and were clearly very limited, and the average functional score based on the Bell scale did indicate that at the beginning of the illness, they were severe.

The duration of illness and functional outcomes were also comparable between both groups, the placebo arm of the immunoglobulin trial as well as the Bell follow up study, which was a defined group for a prolonged period of time.

There was marked consistency in feedback comments. Regaining some control of their life by designing their own management plan was cited by the majority as the most helpful intervention.

### Weaknesses

This study has provided information from only one center and may not be representative of outcomes in another area of the country or internationally. The high rate of post-infective/defined onset CFS has indicated a relatively homogeneous group so that the outcomes may not be applicable to other groups. In addition although the management principles were similar, the actual plan was very individualized and the effective component would not be easy to measure. The timing of the various activity increases and the nature of these activities as well as the timing of psychological assistance was also very varied and individual, based on the young person's situation and readiness both from an illness point of view as well as developmental stage. Similarly, standardized outcome measures that have been used in other studies were not used consistently as the young people did not feel that they were asking questions that were important to them. Similarly school attendance, as such, was not a satisfactory measure of their educational progress but the number of subjects, workload and quality and quantity of task completion was often more satisfactory.

Early in this study it is likely that the presence and significance of orthostatic intolerance may have been missed, although it was noted that those with a slower onset of symptoms (over several months) frequently had associated hyperflexible joints.

Although the regular attempts at formal follow up ensured that the time of recovery was reported close to the event, the proportion of the current sample returned at each time period was not high. As these data were initially collected as part of routine clinical care rather than a formal investigation of duration and predictive factors, the baseline data was not complete. It does however appear that there was little difference in outcomes and other characteristics when both study group and comparison group were part of the final follow up study. So the concerns that lack of completion of baseline data may affect outcomes by potentially reflecting lack of engagement, severity of illness, or not disclosing mental health issues were unfounded.

### Further Study

In order to confirm if these estimates are able to be generalized, this study highlights the need for baseline questionnaire data that is not too onerous to be routinely collected in clinic cohorts. The data needs a severity or frequency scale, and an opportunity to indicate if the symptoms are currently not present, but were previously in the illness, as well as routine documentation of functional improvement and perception of recovery. Confirmation of the impact of depression and anxiety and illness severity at the outset and the perception that some of the contributing factors may be iatrogenic, and focusing on interventions that are important to young people would also be a fruitful area for further investigation. These factors may well vary depending on the sociocultural environment. The overlap between education and health and the importance of remaining engaged in education that was identified by young people as crucial for improved functional outcomes, as well as support and acceptance by family, medical personnel and schools and the importance of regular but not necessarily frequent follow up has been identified as areas that need further study. The impact of management of orthostatic intolerance on improving function warrants further study (17, 40). The suggestions that nonspecific autoimmune responses may be associated and that interventions such as intravenous immunoglobulin may be possible as a treatment option, warrants further investigation in a different cohort.

## Conclusions

Follow up of this cohort indicated that a significant proportion reported recovery and this was confirmed with reported participation in school, work or social activities as well as the global score. The mean duration of illness was 5 years with a range of 1–15 years. Other features of this cohort indicated that there was a high proportion of reported and documented infectious trigger, the most common being EBV. The reported symptoms were very consistent across the group. The ethnic background of the cohort was not representative of the population nor the hospital clientele, and as 17% reported a close family member who had had a prolonged recovery following an infectious illness, had diagnosed ME/CFS in the past or currently, a possible genetic predisposition is likely. Moderate to strongly positive ANA titers were more common than expected and not associated with any other clinical indication of connective tissue disorders. Depression rates were marginally above adolescent baseline rates in Victoria. Higher scores were commonly associated with severity of symptoms, “not being believed” or difficulty negotiating an appropriate program at school. Anhedonia symptoms were rarely reported and these were often when there was a recognized strong family history of depression. There was no difference in outcome rates with those with moderately severe depression at first visit and those with none, however depression was more commonly reported in those with reduced global scores at follow up and those who reported they had not recovered. Additional illnesses were relatively common at follow up and either distinct or comorbid with ME/CFS (Fibromyalgia, anxiety, depression, severe orthostatic intolerance, IBS, fructose/lactose intolerance). There were no suicides reported.

Young people reported that management strategies that allowed them some control back over their lives, that reduced the uncertainty for families and ensured that they received the appropriate understanding and support were the most valuable intervention. Symptom management, especially sleep and headache were very helpful. There were many comments about needing to be believed and understood by the medical profession, teaching profession and family. Comments about alternative therapies usually reflected their parent's anxiety and desire to find something that might help rather than being helpful to the young person.

Many young people reported that they could have improved their self-management as well as acknowledging that they could have been more prepared to accept some help regarding some of the social and psychological issues that they had to face as they were returning back into active life, including dealing with their lack of confidence regarding their ability to cope with adversity or acquisition of social skills due to their absences from school. They acknowledged that this was offered but they were not necessarily ready to access the help.

Remaining engaged in education and therefore their ability to pursue their aspirations was identified as crucially important but involved significant advocacy support and creativity to ensure this occurred. There was a high proportion who reported engaged in or completing post-secondary education (higher than national rate) and more than 95% were working or studying part or full time.

The answers to the common questions are that the majority recover (68% by 10 years). The mean duration was 5 years with range 1–15 years. The functional outcome is generally good, however the duration of illness over such a crucial period of development means that attention to the impact it has on education, social, emotional and physical development was identified by the young people as key to how they coped with, and survived the experience. There was no obvious predictor for recovery at the onset, but there are many helpful interventions including management plan, symptom management and remaining engaged in education. The level of maturity and resilience demonstrated in the young people in the feedback during the follow up was inspiring.

## Author Contributions

The author confirms being the sole contributor of this work and has approved it for publication.

### Conflict of Interest Statement

The author declares that the research was conducted in the absence of any commercial or financial relationships that could be construed as a potential conflict of interest.
