# Supplementary material for: Long Term Follow up of Young People With Chronic Fatigue Syndrome Attending a Pediatric Outpatient Service
Source: Front Pediatr. 2019 Feb 21;7:21. doi: 10.3389/fped.2019.00021 (PMC6393360; doi:10.3389/fped.2019.00021)
Supplement: Supplementary file 1 [file Data_Sheet_1.docx]

**Appendix 1 CHRONIC FATIGUE SYNDROME (ME/CFS)**

**HISTORY RECORD**

**ROYAL CHILDREN'S HOSPITAL.**

DATE

day month year

1. NAME:_________________________________________________________ID ________

2. ADDRESS: _____________________________________________________________

___________________________________________________

3. POSTCODE:______________________________________

4. DATE OF BIRTH: ___________________________

5. SEX (Circle) M / F

6. PHONE NO. (HOME): ____________________________

7. OCCUPATION:__________________________________

8. DATE YOU BECAME ILL Month_______ Year ________

9. DATE of receiving diagnosis Month_______ Year ________

10. FAMILY DOCTOR

(NAME):___________________________________________________________________

(ADDRESS): ________________________________________________________________

_______________________________________________________________

ID

This document is designed to record the details of the symptoms of your illness, particularly the current symptoms, but also those in the past.

*Please answer every question, do not leave any question blank.*

**Read carefully** the scale below, then **score** each of the symptoms listed (Questions 1-38) by ticking () the correct column.

0 **Never** suffer from it.

1 **Mild or rare** symptoms during the **last month**, causing minor disruption to your usual daily activities.

2. **Moderate or frequent** symptoms during the **last month**, causing major disruption to your usual daily activities.

3. **Severe or very frequent** symptoms during the **last month**, making you unable to perform your usual daily activities.

4. Suffered from it **previously** (for a period of at least one month, but not now).

**Symptoms**

|  |  | **0****Never** | **1**  **Mild**  **/rare** | **2**  **Moderate/ frequent** | **3**  **Severe/**  **very frequent** | **4 Previously – not now** |
| --- | --- | --- | --- | --- | --- | --- |
| 1 | Excessive muscle fatigue with minor activity |  |  |  |  |  |
| 2 | Prolonged feeling of fatigue after physical activity (lasting hours or days) |  |  |  |  |  |
| 3 | Muscle pain (not joint pain) after activity |  |  |  |  |  |
| 4 | Muscle pain (not joint pain), even when doing nothing |  |  |  |  |  |
| 5 | Joint pain |  |  |  |  |  |
| 6 | Redness and swelling localised around joints |  |  |  |  |  |
| 7 | Repetitive muscle twitching - on the face |  |  |  |  |  |
| 8 | Repetitive muscle twitching - elsewhere (arms, legs) |  |  |  |  |  |
| 9 | Sudden involuntary jerking of one arm or leg - in sleep |  |  |  |  |  |
| 10 | Sudden involuntary jerking of one arm or leg - when awake |  |  |  |  |  |
| 11 | Headache |  |  |  |  |  |

|  |  | **0****Never** | **1**  **Mild**  **/ rare** | **2**  **Moderate / frequent** | **3**  **Severe/very frequent** | **4 Previously – not now** |
| --- | --- | --- | --- | --- | --- | --- |
| 12 | Nausea |  |  |  |  |  |
| 13 | Stomach pain |  |  |  |  |  |
| 14 | Difficulty swallowing foods |  |  |  |  |  |
| 15 | Recurrent diarrhoea more than 4 loose stools per day |  |  |  |  |  |
| 16 | Repeated fevers and sweats |  |  |  |  |  |
| 17 | Painful, red eye(s) |  |  |  |  |  |
| 18 | Tender glands - in the neck |  |  |  |  |  |
| 19 | Tender glands - elsewhere |  |  |  |  |  |
| 20 | Sore throat (without "common cold" symptoms) |  |  |  |  |  |
| 21 | Feeling of disturbed balance |  |  |  |  |  |
| 22 | Difficulty in focusing vision |  |  |  |  |  |
| 23 | Repeated tingling sensations (fingers, toes or elsewhere) |  |  |  |  |  |
| 24 | Persisting ringing in the ears |  |  |  |  |  |
| 25 | Memory loss |  |  |  |  |  |
| 26 | Loss of concentrating ability |  |  |  |  |  |
| 27 | Difficulty with speech - "lost for the word" |  |  |  |  |  |
| 28 | Palpitations (feeling the heart racing) |  |  |  |  |  |
| 29 | Recurrent chest pain |  |  |  |  |  |
| 30 | Persistent cough |  |  |  |  |  |
| 31 | Shortness of breath with minor activity |  |  |  |  |  |
| 32 | Persistent dryness in the eyes and mouth |  |  |  |  |  |
| 33 | Needing to sleep for long periods |  |  |  |  |  |
| 34 | Disturbed sleep or disrupted sleep pattern |  |  |  |  |  |
| 35 | Vivid dreams or nightmares |  |  |  |  |  |
| 36 | Episode(s) of complete loss of vision in one or both eyes |  |  |  |  |  |
| 37 | Episode(s) of loss of control of the bladder (or bowel) |  |  |  |  |  |
| 38 | Episodes of abrupt anxiety or panic |  |  |  |  |  |

**Tick the single most appropriate response** to the following statements (39-47) with regard to how you have been **in the last month**.

|  |  | **None or a little** | **Some of the time** | **Good part of the time** | **Most of the time** |
| --- | --- | --- | --- | --- | --- |
| 39 | Morning is when I feel the best |  |  |  |  |
| 40 | I have crying spells or feel like it |  |  |  |  |
| 41 | I eat as much as I used to |  |  |  |  |
| 42 | I notice that I am losing weight |  |  |  |  |
| 43 | My heart beats faster than usual |  |  |  |  |
| 44 | I get tired for no reason |  |  |  |  |
| 45 | My mind is as clear as it used to be |  |  |  |  |
| 46 | I find it easy to do things |  |  |  |  |
| 47 | I feel that others would be better off if I were dead |  |  |  |  |

**With regard to the current status of your illness, (during the last month):** Which of the following symptoms (if any) limit your ability to do tasks which you would easily have been able to complete prior to the onset of this illness.

0 ***Never*** *limits my ability.*

1 ***Mild or rare*** *limitation of my ability*.

2 ***Moderate or frequent*** *limitation of my ability.*

3 ***Severe or very frequent*** *limitation of my ability.*

4 *Has caused limitation of my ability* ***in the past, but not now***

|  | ( ) | **0**  **Never** | **1**  **Mild/**  **rare** | **2**  **Moderate / frequent** | **3**  **Severe/ very frequent** | **4**  **In the past / not now** |
| --- | --- | --- | --- | --- | --- | --- |
| 48 | Fatigue |  |  |  |  |  |
| 49 | Muscle or joint pain ( ) |  |  |  |  |  |
| 50 | Concentration difficulties |  |  |  |  |  |
| 51 | Depression ( ) |  |  |  |  |  |
| 52 | Lack of motivation |  |  |  |  |  |

**With regard to the onset of your illness**:

53 Did the illness appear to follow (within one month):

*0 Came on without any precipitant*

*1 An operation (with a general anaesthetic)*

*2 A vaccination*

*3 A viral or "flu-like" illness*

*4 A specific infection, diagnosed by blood tests* ( )

54 In what month and what year did the illness develop ____/____

**With regard to the initial illness (if present):**

Please tick the appropriate response

|  | **No** | **Unsure** | **Yes** |
| --- | --- | --- | --- |
| 55 Was glandular fever (EBV) proven (blood tests)? |  |  |  |
| 56 Was any other specific infection proven (blood tests) |  |  |  |

57 What was the infection? _______________________________________________

**With regard to your family:**

|  | **No** | **Unsure** | **Yes** |
| --- | --- | --- | --- |
| 58 Is there a family history of thyroid disease or goitre |  |  |  |
| 59 Are there other family members with a similar disorder or with known Chronic Fatigue Syndrome (ME)? |  |  |  |

60 **With regard to the pattern of the illness:**

Which of the following best describes the pattern of the symptoms of your illness, since its onset:

0 *Relapsing and remitting (i.e. periods of complete recovery, then recurrence).*

1 *Continuous in the past, now relapsing*.

2 *Relapsing in the past, now continuous*.

3 *Continuous, but with fluctuating severity.*

4 *Continuous, at the same level of severity*. ( )

61 **With regard to the frequency of common colds (runny nose, sneezing etc.)**

Do you experience common colds:-

0 ***No more, or less*** *frequently than prior to the illness*

1 ***Less frequently*** *than prior to the illness*

2 ***More frequently*** *than prior to the illness* ( )

62 **With regard to the impact of this illness in your life:**

Describe your ability to participate in various aspects of life that may be affected because of this illness, using the scale below:

0 ***Not reduced****, unchanged since before the illness*

1 ***Mildly reduced***

2 ***Moderately reduced***

3 ***Completely unable*** *to participate*

4 *Reduced in the past, but not now*

Your work/school ( )

Your social life ("going out", parties, etc.) ( )

Your recreation (including sport) ( )

63 Describe **the effect of your illness on your relationship with your family**, using the scale below:-

0 *No effect at all, or beneficial effect*

1 ***Mildly*** *damaging effect*

2 ***Moderately*** *damaging effect*

3 ***Severely*** *damaging effect*

4 *No effect now, but damaging effect* ***in the past***

( )

64 **With regard to your work/school** (circle)

Are you currently there full-time part-time not attending

Do you use the Visiting teacher services Yes / No

Do you use the Correspondence School Yes / No

65 **With regard to the medical care you have received for this illness**:

How many visits to doctor(s) did you make, regarding this illness:-

In the last 12 months? ( )

In the first 12 months of the illness? ( )

66 Which of the following **best** describes your current level of physical **ability** (in the last month):

0 *Physical ability unchanged from before the illness*

1 *Able to do vigorous work or exercise but reduced capacity*

2 *Able to walk without limitation, including up stairs*

3 *Able to walk for short distances outside the house only*

4 *Able to walk around the house but not to leave it*  ( )

67 Describe your current level of regular physical **activity** on the following scale:

0 *No regular exercise*

1 *No active exercise but walk regularly*

2 *One hour per week of active work or exercise (e.g. digging, heavy lifting, swimming, tennis)*

3 *One to four hours per week of active work or exercise*

4 *One or more hours daily of active work or exercise*

Prior to the onset of this illness ( )

Currently (in the last month) ( )

68 Mark the number of hours per day currently that you would usually spend in the activities listed, using the scale below:

0 *Less than four hours*

1 *Four to eight hours*

2 *Eight to twelve hours*

3 *Twelve to sixteen hours*

4 *More than sixteen hours*

Sleeping (at night) ( )

Sleeping (during the day) ( )

Lying or sitting down (not sleeping) ( )

*69 Mark the number of hours per day currently that you would usually spend in the activities listed, using the scale below:*

0 *Less than one half an hour* 3 *Two to three hours*

1 *One half to one hour*4 4 *More than three hours*

2 *One to two hours*

Doing sedentary activity (e.g. writing, reading) ( )

Mobile activity (walking, light daily chores, standing ( )

Vigorous activity (running, lifting, (heavy daily chores) ( )

**Which level on the CFIDS Disability Scale describes (a) your present level of health and (b) your most pronounced ill health**

(a) Present level of health ____________

(b) Most pronounced ill health ____________

**CFIDS Disability Scale**

10 No symptoms at rest. No symptoms with exercise

Normal overall activity level

Able to work full time without difficulty

9 No symptoma at rest. Mild symptoms with activity.

Normal overall activity level.

Able to work full time without difficulty.

8 Mild symptoms at rest. Symptoms worsened by exertion.

Minimal activity restriction noted for activities requiring exertion only.

Able to work full time with difficulty in jobs requiring exertion.

7 Mild symptoms at rest. Some daily activity limitation clearly noted.

Overall functioning close to 90% of expected except for activities requiring exertion.

Able to work full time with difficulty.

6 Mild to moderate symptoms at rest. Daily activity limitation clearly noted.

Overall functioning 70% to 90% of expected except for activities requiring exertion.

Able to work full time with difficulty.

5 Moderate symptoms at rest. Moderate to severe symptoms with exercise or activity; overall activity level reduced to 70% of expected.

Unable to perform strenuous duties, but able to perform light duty or desk work 4-5 hours

a day, but requires rest periods.

4 Moderate symptoms at rest. Moderate to severe symptoms with exercise or activity, overall activity level reduced to 50% to 70% of expected. Not confined to house. Unable to perform strenuous duties; but able to perform light duty or desk work 2-3 hours a day, but requires rest periods.

3 Moderate to severe symptoms at rest. Severe symptoms with any exercise. Overall activity level reduced to 50% of expected. Usually confined to house. Unable to perform any strenuous duties. Able to perform desk work 2-3 hours a day, but requires

rest periods.

2 Moderate to severe symptoms at rest. Unable to perform strenuous activity. Overall activity level reduced to 30% to 50% of expected. Unable to leave the house except rarely. Confined to bed most of the day; Unable to concentrate for more than an hour a

day.

1 Severe symptoms at rest.

Bed ridden the majority of the time. No travel outside of the house. Marked cognitive symptoms preventing concentration.

0 Severe symptoms at rest.

Bed ridden constantly. Unable to care for self.

(Reference: Dr David S Bell, The disease of a thousand names. 1991, Pollard Publications, New York)

**Appendix 2**  **Follow up Questionnaire**

Date: / /

Date of birth / / Name:_________________ID ____

Please tick () the appropriate response.

1 Are you attending school/tertiary education/work full time, ❑

more than half time, ❑

less than half time? ❑

2 Did you, or are you, completing a tertiary course? yes ❑ no ❑

If so, which course/s

1. If you are working – what is the nature of your work?

_____________________________________________________________

4 If you are not at school full time, have you used the Visiting Teacher Services since you were sick? yes ❑ no ❑

5 Are you still using them? yes ❑ no ❑

6 Are you enrolled with Distance Education? yes ❑ no ❑

7 Do you receive the Disability Support Pension? yes ❑ no

8 Do you feel you are no longer suffering from CFS? yes ❑ no ❑

9 How would you rate yourself out of 10? ❑❑/10

(1=bedridden, 10=well)

10 When was the onset of CFS? ❑❑ / ❑❑

Month Year

11 How long had you suffered from CFS before it was diagnosed?❑❑ months

12 How long had you suffered from CFS before receiving help? ❑❑ months

13 If you have recovered, how long did the illness last? ❑❑ months

14 Have you had significant infection/s in the last twelve months?

yes ❑ no

If so, how many? ❑❑

How long were you unwell on average? ❑❑ weeks

Did any symptoms of CFS recur? yes ❑ no ❑

15 Have you had any prolonged recurrence of symptoms? yes ❑ no ❑

If yes - How long did the symptoms last? ❑❑ weeks

Was there any particular or obvious trigger? yes ❑ no ❑

If so – what was it?

16 Have you had any other serious illness? yes ❑ no ❑

If so – what was / is it?

17 Have you found any professionals helpful? yes ❑ no ❑

If so - which ones? __________________________________________________

In what way?

18 Has any information been particularly helpful/useful? yes ❑ no ❑

If so - which? _______________________________________________________

19 Is there anything during your illness which could have been handled better or differently?

yes ❑ no ❑

If so - What?

20 Have you sought alternative treatments in the management of your illness?

yes ❑ no ❑

If so - What were they? _________________________________________________

_____________________________________________________________________

_____________________________________________________________________

Do you feel they have worked? yes ❑ no ❑

21 Do you have any other members of your family who have (or still have CFS)

yes ❑ no ❑

If so, what relationship? eg., sibling (brother or sister), mother, father, mother’s brother, or cousin (on father’s side etc)

_____________________________________________________________________

22 Any other comments? ___________________________________________________

Thank you so much!

**Which level on the CFIDS Disability Scale (Bell) describes**

(a) Present level of health ____________

(b) Most pronounced ill health ____________

**CFIDS Disability Scale**

10 No symptoms at rest. No symptoms with exercise. Normal overall activity level

Able to work full time without difficulty

9 No symptoms at rest. Mild symptoms with activity.

Normal overall activity level. Able to work full time without difficulty.

8 Mild symptoms at rest. Symptoms worsened by exertion.

Minimal activity restriction noted for activities requiring exertion only.

Able to work full time but with difficulty in jobs requiring exertion.

7 Mild symptoms at rest. Some daily activity limitation clearly noted.

Overall functioning close to 90% of expected except for activities requiring exertion.

Able to work full time with difficulty.

6 Mild to moderate symptoms at rest. Daily activity limitation clearly noted.

Overall functioning 70% to 90% of expected except for activities requiring exertion.

Able to work full time with difficulty.

5 Moderate symptoms at rest. Moderate to severe symptoms with exercise or activity;

overall activity level reduced to 70% of expected.

Unable to perform strenuous duties, but able to perform light duty or desk work 4-5 hours

a day, but requires rest periods.

4 Moderate symptoms at rest. Moderate to severe symptoms with exercise or activity,

overall activity level reduced to 50% to 70% of expected. Not confined to house.

Unable to perform strenuous duties; but able to perform light duty or desk work 2-3 hours

a day, but requires rest periods.

3 Moderate to severe symptoms at rest. Severe symptoms with any exercise. Overall activity level reduced to 50% of expected. Usually confined to house. Unable to perform any strenuous duties. Able to perform desk work 2-3 hours a day, but requires rest periods.

2 Moderate to severe symptoms at rest. Unable to perform strenuous activity. Overall activity level reduced to 30% to 50% of expected. Unable to leave the house except rarely. Confined to bed most of the day; Unable to concentrate for more than an hour a day.

1 Severe symptoms at rest.

Bed ridden the majority of the time. No travel outside of the house. Marked cognitive

symptoms preventing concentration.

0 Severe symptoms at rest.

Bed ridden constantly. Unable to care for self.

(Ref: David S Bell, The disease of a thousand names. 1991, Pollard Publications, New York)

**Appendix 3: Young people’s global functional scale for ME/CFS**

| Score | School/work | Stamina | Recovery | Social | Symptoms |
| --- | --- | --- | --- | --- | --- |
| **10** | Full time  If study – often part time job as well | ‘normal’ | Recovers well | Active social life | No residual symptoms. Feels ‘back to normal’ and comparable to peers. |
| **9** | Full time – often with part time job as well | Gets more tired than would expect | Can participate in active sport | Good social life | Manages well but needs to ‘pace’ |
| **8** | Full time – either work or study not both especially if playing some sport as well | Needs occasional day to ‘recover’ | Needs some time to recover after vigorous activity | Good social life | Has periods with some symptoms and rarely feels ‘refreshed’ |
| **7** | Full time either work or study | Reduced load | Some regular sport but usually not vigorous | Some social contact | Continuous symptoms but not severe. Rarely feels ‘well’ but not prolonged recovery time |
| **6** | Part time | Reduced load | Infrequent active, regular mild activity eg walking | Reduced social contact and needs to intentionally keep in touch. | Has to carefully plan activity and rest. Symptoms present continuously with fluctuating severity |
| **5** | Less than half time | Reduced subject load if studying | Regulates activity – no vigorous activity | Regulated in timing and quantity | Occasionally can attend activity with recovery period – persistent symptoms |
| **4** | Few hours per week concentrating | Significantly reduced load if studying – both subject and content | Minimal regular activity | Major impact on life | Intrusive symptoms that worsen markedly after activity |
| **3** | Minimal participation | Only for short periods across the week | Struggles with activities of daily living | Reduced | Persistent and intrusive |
| **2** | Attempting to read but difficulty with recall | Very limited stamina | Limited outside of home. Modified activities of daily living | Markedly Reduced | Persistent and intrusive |
| **1** | Not participating | Very limited stamina | Rarely leaving home. Needing assistance with daily activities | Very limited | Constant moderate to severe symptoms |
